# An artificial intelligence-based ecological index for prognostic evaluation of colorectal cancer

**DOI:** 10.1186/s12885-023-11289-0

**Published:** 2023-08-17

**Authors:** Qicong Chen, Ming Cai, Xinjuan Fan, Wenbin Liu, Gang Fang, Su Yao, Yao Xu, Qian Li, Yingnan Zhao, Ke Zhao, Zaiyi Liu, Zhihua Chen

**Affiliations:** 1 Institute of Computing Science and Technology, Guangzhou University, No. 230, Outer Ring West Road, Guangzhou, 510006 China; 2 Guangdong Provincial Key Laboratory of Artificial Intelligence in Medical Image Analysis and Application, Guangzhou, 510080 China; 3 Department of Radiology, Guangdong Provincial People’s Hospital (Guangdong Academy of Medical Sciences), Southern Medical University, Guangzhou, 510080 China; 4 Department of Pathology, Sixth Affiliated Hospital of Sun Yat-Sen University, Guangzhou, China; 5 Department of Pathology, Guangdong Provincial People’s Hospital, Guangdong Academy of Medical Sciences, Guangzhou, China; 6 Guangdong Provincial People’s Hospital, Guangdong Cardiovascular Institute, Guangdong Academy of Medical Sciences, Guangzhou, China

**Keywords:** Colorectal cancer, Ecological index, Artificial intelligence, Nuclei segmentation, Spatial association, Digital pathology

## Abstract

**Background and objective:**

In the tumor microenvironment (TME), the dynamic interaction between tumor cells and immune cells plays a critical role in predicting the prognosis of colorectal cancer. This study introduces a novel approach based on artificial intelligence (AI) and immunohistochemistry (IHC)-stained whole-slide images (WSIs) of colorectal cancer (CRC) patients to quantitatively assess the spatial associations between tumor cells and immune cells. To achieve this, we employ the Morisita-Horn ecological index (Mor-index), which allows for a comprehensive analysis of the spatial distribution patterns between tumor cells and immune cells within the TME.

**Materials and methods:**

In this study, we employed a combination of deep learning technology and traditional computer segmentation methods to accurately segment the tumor nuclei, immune nuclei, and stroma nuclei within the tumor regions of IHC-stained WSIs. The Mor-index was used to assess the spatial association between tumor cells and immune cells in TME of CRC patients by obtaining the results of cell nuclei segmentation. A discovery cohort (*N* = 432) and validation cohort (*N* = 137) were used to evaluate the prognostic value of the Mor-index for overall survival (OS).

**Results:**

The efficacy of our method was demonstrated through experiments conducted on two datasets comprising a total of 569 patients. Compared to other studies, our method is not only superior to the QuPath tool but also produces better segmentation results with an accuracy of 0.85. Mor-index was quantified automatically by our method. Survival analysis indicated that the higher Mor-index correlated with better OS in the discovery cohorts (HR for high vs. low 0.49, 95% CI 0.27–0.77, *P* = 0.0014) and validation cohort (0.21, 0.10–0.46, < 0.0001).

**Conclusion:**

This study provided a novel AI-based approach to segmenting various nuclei in the TME. The Mor-index can reflect the immune status of CRC patients and is associated with favorable survival. Thus, Mor-index can potentially make a significant role in aiding clinical prognosis and decision-making.

## Introduction

Colorectal cancer (CRC) is the third most common cancer worldwide and the second fatal cancer globally. The observed increase in CRC incidence and mortality will last in the coming decades [1]. Tumor cells and tumor microenvironments (TME) jointly limit and influence the occurrence and development of tumors in CRC patients. TME is closely related to the progression of tumor metastasis and patients' response to treatment [2]. Specifically, it harbors non-immune cells, immune cells, and tumor cells which influence the development and progression of cancer through interaction with surrounding cells. In this complex ecosystem, these various cell types interact to determine the tumor progression [3, 4].

The information in immunohistochemical (IHC)-stained whole-slide images (WSIs) including cell density, cell type, and site of immune cells is proved to be valuable prognostic tool [5]. T cells play a critical role in the body's defense against infections and cancer, with their infiltration in human cancer being indicative of immune recognition [6]. Among T cell subsets, the total lymphocyte count (CD3 +) and specific subtypes (CD4 + , CD8 +) have strong associations with the survival outcomes of CRC patients. CD8 + T lymphocytes, in particular, are essential anti-tumor immune cells that directly eliminate tumor cells by releasing cytotoxic molecules. The density of CD3 + T lymphocytes, reflecting the overall functionality of the immune system within the tumor microenvironment (TME), serves as an important prognostic indicator. Notably, the density of CD3 + T cells has shown a stronger correlation with patient survival outcomes compared to cytotoxic T cells (CD8 + T cells) [4, 7].

Previous research has established that the tumor ecosystem is highly complex and heterogeneous. In this ecosystem, the interaction of tumor cells, immune cells, and their microenvironment have profound effects at all stages of CRC disease [8]. It has been demonstrated that the spatial distribution of different immune cells amongst the immunological data provides the most effective information for breast cancer research [9]. Maley et al. (2015) evaluated the results of haematoxylin and eosin (HE) staining sections from 1002 breast cancer patients and found that co-localization of cancer cells and immune cells is an independent predictor of survival. The new predictor was evaluated using the Morisita-Horn ecological index (Mor-index). It has been proved the new predictor can be fully reproducible and have a more robust predictive performance than other standard clinical variables [10]. A great deal of previous research into CRC patients has explored the effects of immune cell density and tumor cell density on cancer prognosis and prediction [11–14]. However, few studies have quantified the relationship between spatial patterns of immune cells and cancer cells.

In recent years, there has been rapid development in artificial intelligence (AI), which has found extensive applications in the management and analysis of large biomedical datasets, aiding in diagnosis and clinical decision-making. Particularly, deep learning technology has shown promise in early tumor identification and enhancing the efficiency of tissue classification in CRC. The utilization of AI technology offers notable advantages in terms of high efficiency and reproducibility, enabling efficient nuclei segmentation and facilitating further investigations into cell relationships [15, 16].

This study aims to employ AI methods for the precise segmentation of immune nuclei, stroma nuclei, and tumor nuclei in immunohistochemically stained whole-slide images (WSIs) of CRC. By identifying and locating these three cell types, the spatial associations and interactions between immune cells and tumor cells can be explored. Additionally, the prognostic significance of the Mor-index in CRC was investigated.

## Methods

### Patients

The study included clinical data of 569 patients from two centers, the Guangdong Provincial People's Hospital (GDPH) and the Sixth Affiliated Hospital of Sun Yat-sen University (SYSU6). Our study recruited patients with colorectal cancer confirmed stage I–III CRC who has been approved by the related institutional review board. 432 patients from GDPH were enrolled in the discovery cohort and 137 patients from SYSU6 were enrolled in the validation cohort. Exclusion criteria: stage IV CRC patients, WSIs with poor pathological image quality that interfere with observation, and sections with missing clinical information. Clinical and follow-up information in regard to the patient including age, TNM stage and tumor site is obtained from electronic medical records. The clinical endpoint of this study was overall survival (OS, time from the resection to date of death of any course).

## IHC staining and whole-slide images acquisition

Human anti-CD3 is an excellent marker for T cell detection due to its specific binding to T cells. The immune status was assessed and quantified using immunohistochemistry (IHC) slides. The detailed process of IHC slides can be found in one of our previous studies [17]. The IHC slides were scanned into digitized IHC-stained WSIs by the scanner (Leica, Aperio-AT2, and GT450, USA) at a 40** × **magnification (0.25–0.26 µm/pixel).

### Tissue segmentation

**Fig. 1:**
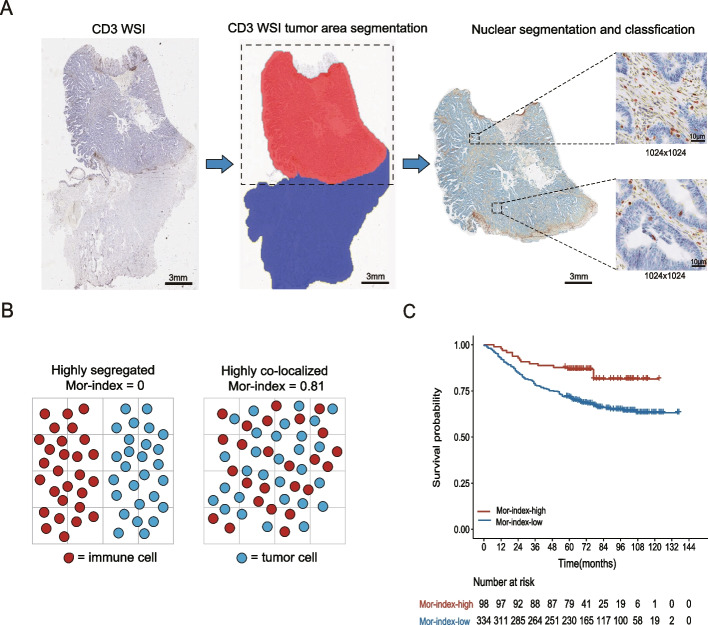
Nuclei segmentation based on deep learning technology and traditional computer segmentation measures the spatial association of tumor cells and immune cell. **A** Tissue segmentation is performed using a CNN (VGG-19) model to get the tumor region in WSI, the blue part is the tumor region to be preserved, and the red part is the cropped part. After that, nuclear segmentation and classification in the tumor area. The red boundaries represent the nuclei of immune cells, the blue boundaries represent the nuclei of tumor cells, and the yellow boundaries represent the nuclei of stroma cells in the image patch (1024 × 1024 pixel^2^ at 40 ×). **B** A schematic diagram on any spatial plane showing Mor-index how common domain statistics distinguish between highly segregated cell patterns and highly co-localized cell patterns. **C** Kaplan–Meier curve predicting survival probability in the discovery cohort. CNN, convolutional neural network; WSI, whole-slide image; IHC, immunohistochemical; Mor-index, Morisita-Horn ecological index

A convolutional neural network (VGG-19) was used to segment tissues in IHC-stained WSIs automatically. By selecting the tissue category with the highest probability of prediction as to the prediction category, nine tissue categories (stroma, tumor epithelium, lymphocyte, mucous, normal mucosa, adipose, debris, smooth muscle, and background) were separated. The detailed steps can be referred to in our previous research [18]. The regular tissue area was acquired by combining adipose and muscle area. Since this study only quantified the spatial relationship of cell in the tumor area, the tumor area was reserved for analysis. The tumor area was obtained by merging debris, tumor stroma, and tumor epithelial areas. The flowchart of the entire study is shown in Fig. 1. For visualization, tumor area is represented in red, and regular tissue area is represented in blue.

### Immune, tumor and stroma nuclei segmentation

Nuclei segmentation was performed in the WSI for classifying immune nuclei, tumor nuclei, and stroma nuclei. The tumor area, which tiled into a large number of image patches (1024** × **1024 pixel^2^ at 40** × **magnification), was obtained by tissue segmentation. Subsequently, a Gabor filter was used for texture analysis of each image patch, and its features were extracted and segmented to obtain tumor epithelial regions. The following is a detailed description of the segmentation steps.

Firstly, an array of Gabor filters with different frequencies and orientations is designed to localize different, roughly orthogonal, subsets of frequency and orientation information in the input image [19]. Regularly sample orientations between [30,180] degrees in steps of 30 degrees, the sampling wavelength is 2. The image patch is processed by the Gabor filter to obtain a Gabor magnitude image. 30 degrees to 180 degrees can cover various texture orientations that may exist in IHC-stained WSIs. The wavelength of 2 is well suited for capturing fine cellular structures and texture features. The step size of 30 degrees can somewhat balance computational efficiency and detail preservation.

Next, the Gabor magnitude image is transformed into Gabor features through a series of pre-processing. This pre-processing includes Gaussian smoothing, adding extra spatial information to the feature set, and bringing our feature set into the format required by the K-means function. Set the maximum number of iterations of the K-means function to 5 to limit the iteration count allows the algorithm to converge to an acceptable clustering result faster, and set the number of clusters to 2 to segment the image into 2 regions. Then, the K-means clustering algorithm is used to classify Gabor texture features into two categories to get the white region of interest (ROI) and the black background. The ROI area underwent morphological operations such as swelling and corrosion to obtain the tumor epithelial areas.

Finally, the previously developed nuclei segmentation software was utilized to segment tumor nuclei specifically in the tumor epithelial areas. For immune nuclei segmentation, the Bernsen algorithm was employed with a scan frame size of 77 pixels and a contrast threshold of 15. The remaining nuclei were classified as stroma nuclei. The choice of a window size of 77 pixels was based on its suitability for capturing the size range of most immune nuclei. Immune nuclei, which typically exhibit a brown appearance, generally possess local thresholds exceeding 15, thus justifying the selection of a contrast threshold of 15. As a result, three distinct types of nuclei were successfully separated and quantified. In Fig. 1A, the boundaries of immune cells are depicted in red, tumor cells in blue and stroma cells in yellow.

The accuracy of our cell nuclei segmentation model is validated using three evaluation criteria. True positive (TP) represents the number of correct positive predictions. False positive (FP) represents the number of incorrect positive predictions. True negative (TN) represents the number of correct negative predictions. False negative (FN) represents the number of incorrect negative predictions. Accuracy is calculated as the ratio of correct predictions to the total number of predictions.

$$Accuracy = \frac{TP + TN}{{TP + TN + FP + FN}}$$

The Dice coefficient is an ensemble similarity measure, usually used to calculate the similarity of two samples.

$$Dice = \frac{{{2}TP}}{2TP + FP + FN}$$

The Mean Intersection over Union (MIoU) is the standard metric for semantic segmentation and computes the average of the ratios of the intersections and unions of all categories.

$$\begin{gathered} MI{\text{o}}U = \frac{1}{k + 1}\sum\limits_{{{\text{i}} = 0}}^{k} {\frac{TP}{{FN + FP + TP}}} \hfill \\ \hfill \\ \end{gathered}$$

### Measuring spatial association between tumor cell and immune cell

The Morisita-Horn index, originally proposed by Horn as an ecological measure, has proven to be a valuable tool for studying community structure and analyzing data such as diet preferences and habitat preferences in ecology [20, 21]. In the context of our study, this index is employed to quantify the spatial colocation of immune cells and tumor cells, providing insights into their spatial associations within the tumor microenvironment. This index can be calculated based on the the square tessellation method or the Voronoi method. For square tessellation, a fixed square size h-by-h was used for all tumor areas. H = 1024 pixel was used as the fixed square length and tumor area were divided into squares of size 1024** × **1024 pixel^2^, which was defined as polygon i. The study used the square tessellation method, where each polygon i is an image patch (1024** × **1024 pixel^2^ at 40** × **magnification). The number of immune nuclei and tumor nuclei of each polygon i was obtained as ${\mathrm{n}}_{\mathrm{i}}^{\mathrm{l}}$ and ${\mathrm{n}}_{\mathrm{i}}^{\mathrm{c}}$, respectively. In the following data analysis, polygons with low nuclei density were excluded. The excluded low-density polygon was defined as $\frac{{\mathrm{n}}_{\mathrm{i}}}{{\mathrm{d}}_{\mathrm{i}}}\le 0.$ 0002 of polygon i, where ${\mathrm{n}}_{\mathrm{i}}$ represented the total number of nuclei and ${\mathrm{d}}_{\mathrm{i}}$ represented the pixel size of polygon i. To measure the spatial correlation between tumor cells and immune cells, the number of immune nuclei and tumor nuclei of polygon i was input firstly, and then calculated the Morisita-Horn similarity index. The proportion of immune nuclei and tumor nuclei in polygon i are represented by ${\mathrm{p}}_{\mathrm{i}}^{\mathrm{l}}$ and ${\mathrm{p}}_{\mathrm{i}}^{\mathrm{c}}$ respectively.i.e.

$${p}_{i}^{l}=\frac{{n}_{i}^{l}}{{\sum }_{i}{n}_{i}^{l}}\ {p}_{i}^{c}=\frac{{n}_{i}^{c}}{{\sum }_{i}{n}_{i}^{c}}$$

Morisita-Horn's tumor and immune cell community structure similarity index can be expressed by this formula:

$$Mor-index=\frac{2{\sum }_{i}{p}_{i}^{l}{p}_{i}^{c}}{{\sum }_{i}{({p}_{i}^{l})}^{2}+{\sum }_{i}{({p}_{i}^{c})}^{2}}.$$

The Mor-index is used to compare the degree of co-localization of immune and tumor cells. As shown in Fig. 1B, Mor-index is equal to 0 indicates that there is a highly segregated between immune cells and tumor cells, and 0.81 indicates that immune cells and tumor cells are highly co-localized.

### Statistical analysis and software

Image pre-processing steps, including tissue segmentation, nuclei segmentation, nuclei counting, and other necessary pre-processing tasks, were performed using MATLAB (R2019a, MathWorks, USA). Kaplan–Meier curves were utilized to stratify patients, and differences between patient groups were evaluated using log-rank tests, with statistical significance defined as *P* < 0.05. Univariate and multivariate analyses were conducted using Cox proportional hazard regression models to analyze patient data. Hazard ratios (HRs) with 95% confidence intervals (CIs) were calculated using the Cox model. Model accuracy (ACC) was used to describe the classification accuracy, while the Dice coefficient and the mean intersection over union (MIoU) were employed to assess segmentation performance. Statistical analysis was carried out using R language packages such as survival, survminer, ggplot2, ggpubr, ggpmisc, ggforest, Hmisc, and rms.

### Patients

The 569 CRC patients were recruited for this study, with a slightly higher number of patients older than 60 years than those younger (224 vs. 345). Basic clinicopathological information can be seen in Table 1, including age, sex, TNM stage, tumor site. 69 (interquartile range [IQR], 67 − 76) months was the median follow-up time of the discovery cohort and 63 (IQR, 76 − 89) months was the median follow-up time of the validation cohort. Besides, with the increase in follow-up time of 1, 3, and 5 years, the survival rate decreased gradually. The percentage of survival rate was from 92.9% to 74.4% in the discovery cohort and from 94.1% to 82.2% in the validation cohort. Significant differences were found between the two cohorts on tumour site.

**Table 1:** Distribution of clinicopathological features in discovery and validation cohorts

	**Discovery cohort** **(128/432)**^**a**^	**Validation cohort** **(25/137)**^**a**^	**P**
**Age**			0.121
≤ 60 years	161 (38.6%)	63 (46.0%)	
> 60 years	271 (62.4%)	74 (54.0%)	
**Sex**			0.743
Male	256 (59.3%)	84 (61.3%)	
Female	176 (40.7%)	53 (38.7%)	
**T**			0.248
T1	11 (2.5%)	8 (5.8%)	
T2	64 (14.8%)	36 (26.3%)	
T3	317 (73.4%)	90 (65.7%)	
T4	40 (9.3%)	3 (2.2%)	
**N**			0.127
N0	231 (53.2%)	87 (63.5%)	
N1	125 (28.9%)	36 (26.3%)	
N2	77 (17.8%)	14 (10.2%)	
**Stage**			0.049
I	59 (13.7%)	39 (28.5%)	
II	172 (39.8%)	47 (34.3%)	
III	201 (46.5%)	51 (37.2%)	
**Tumour site**			< 0.001
Colon	257 (59.5%)	0 (0%)	
Rectum	175 (40.5%)	137 (100%)	
**MSI status**			< 0.001
MSI	31 (7.1%)	0(0%)	
MSS	265 (61.3%)	48(35.0%)	
NA	136(31.6%)	89(65.0%)	

*Abbreviation: CI* confidence interval, *MSI* microsatellite instability, *MSS* microsatellite stability, *NA* not available, *NA* not available

*P* value was performed by Kruskal–Wallis or χ^2^ test where appropriate

^a^Numbers in parentheses are number of events/total number of patients

### Accuracy of cell nucleus segmentation results

**Fig. 2:**
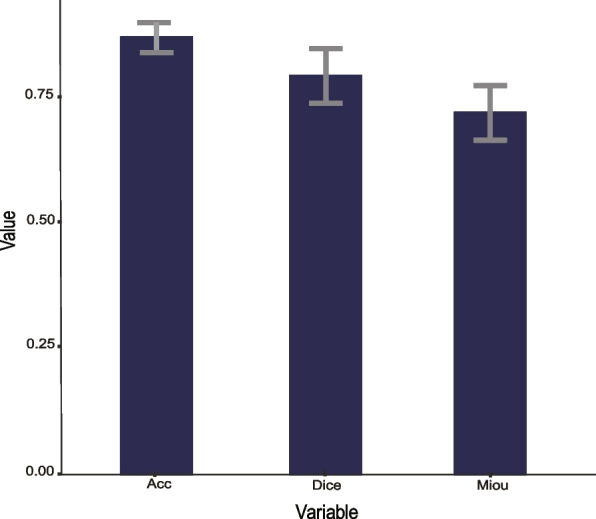
The results of the three calculation indicators of the model are Acc, Dice, and Miou in this study. Acc, accuracy; Dice, dice coefficient; Miou, the Mean Intersection over Union

**Fig. 3:**
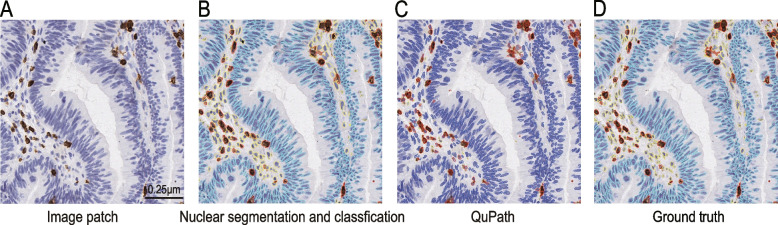
The red nucleus boundaries are immune nucleus, the blue nucleus boundaries are tumor nucleus, and the yellow nucleus boundaries are stroma nucleus. **A** An image patch (512 × 512 pixel^2^ at 40 ×). **B** The nuclei segmentation based on our method. **C** The nuclei segmentation based on QuPath. **D** Ground truth

Twenty ground truths (512** × **512 pixel^2^ at 40 **×**) were used as a validation set to validate the nuclei segmentation result of our method. The average accuracy of the nuclei segmentation results is excellent at 0.85 and the standard deviation was 0.03. The Dice coefficient and the MIoU of this automated cell nuclei classification model were 0.79 and 0.74, respectively, with the same standard deviation being 0.05 (Fig. 2). We also used QuPath (v0.3.2), the open source software for biological-image analysis, for nuclei segmentation of this validation set. The Dice coefficient and the MIoU were only 0.35 and 0.5 respectively, and the average accuracy of the nuclei segmentation results was only 0.75. The standard deviations of the three calculation indicators wegeneralization re 0.07, 0.04, and 0.05, respectively. Compared with our method, although QuPath supports multiple classifications of nuclei, the of this algorithm is worse (Fig. 3).

### Association between Mor-index and CD3^+^ cell infiltration density, MSI and MSS

**Fig. 4:**
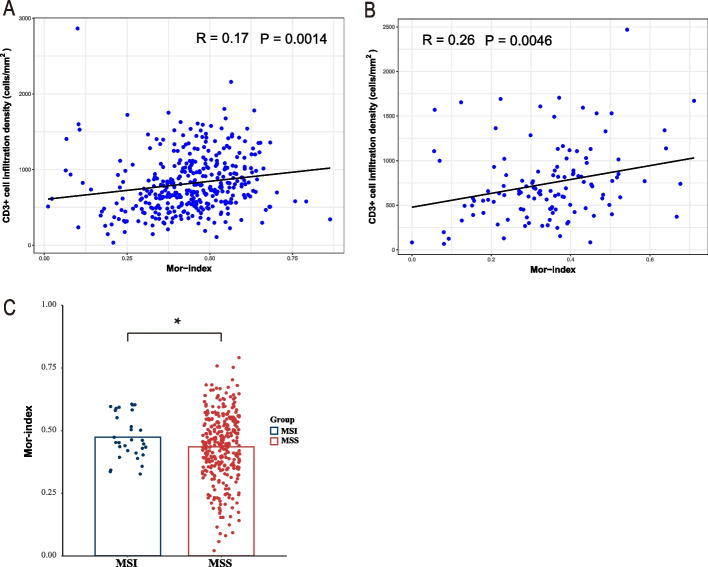
Correlation between Mor-index and CD3^+^ cell infiltration, MSI, and MSS. **A** The correlation analysis between Mor-index and CD3^+^ cell infiltration density in the discovery cohort (*R* = 0.17, *P* = 0.0014). **B** The correlation analysis between Mor-index and CD3^+^ cell infiltration density in the validation cohort (*R* = 0.26, *P* = 0.0046). **C** Student’s t-test was used to compare the distribution of Mor-index among different types of microsatellite status. (*P < 0.05, Student’s t-test). Mor-index, Morisita-Horn ecological index; MSI microsatellite instability; MSS, microsatellite stability

To evaluate whether there could be a correlation between the Mor-index with CD3^+^ cell infiltration density, microsatellite instability (MSI) and microsatellite stability (MSS), additional analyses were performed. In the discovery cohort, a correlation analysis between Mor-index and CD3^+^ cell infiltration density was conducted, and it revealed a weak correlation between the two variables (Pearson *R* = 0.17, *P* = 0.0014, Fig. 4A). This finding was validated in the validation cohort (Pearson *R* = 0.26, *P* = 0.0046, Fig. 4B). Due to the validation cohorts consisted of data from the rectum and all the data in the validation cohort were in an MSS status., so the validation cohorts and the discovery cohorts were merged. Figure 4C shows the distribution of Mor-index among different types of microsatellite status. The MSI group had a higher mean Mor-index than the MSS group, with a statistical difference (*P* < 0.05).

### Prognostic value of the Mor-index

**Fig. 5:**
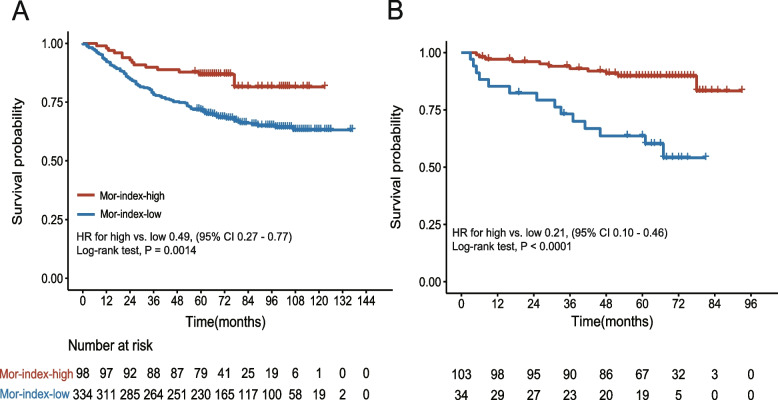
Immune-tumor cell colocation predicts long term outcomes in CRC patients. Kaplan–Meier curves analysis for Mor-index-high and Mor-index-low patients. **A** Mor-index in the discovery cohort. **B** Mor-index in the validation cohort. CRC, colorectal cancer; Mor-index, Morisita-Horn ecological index

The tumor region in the IHC-stained WSIs of all patients was segmented, and then the immune and tumor nuclei were segmented to calculate the Mor-index by the number of cell nuclei. The continuous Mor-index was divided into two groups with 50% as the cutoff. A total of 98 cases (22.7%) were divided into the Mor-index-high group and 334 cases (77.3%) into the Mor-index-low group. Survival curves of the high and low groups are represented in Fig. 5A. The Kaplan–Meier curve suggested that the high and low groups were significantly different in predicting OS, and the high group is associated with favorable OS. In the discovery cohort, OS was significantly better for patients with high. The 5-year survival rates were 72% in the low group and 87% in the high group (*P* = 0.0014; Fig. 5A). Patients with high and low Mor-index experienced a significant difference in survival (unadjusted HR 0.49, 95% CI 0.27–0.77; *P* = 0.003; Table 2). In the validation cohort, these findings were confirmed (0.21, 0.10–0.46, < 0.001; Table 2), and the 5-year survival rates were 64% in the low group and 90% in the high group (Fig. 5B).

**Table 2:** Univariate analysis including age, sex, stage, tumour site, and Mor-index for OS in the two cohorts

	**Discovery cohort** **HR (95% CI)**	**P**	**Validation cohort** **HR (95% CI)**	**P**
**Age**	1.03 (1.02–1.05)	< 0.001	1.04 (1.00–1.08)	0.029
**Sex**				
Male	1			
Female	1.04 (0.73–1.48)	0.824	0.74 (0.32–1.72)	0.490
**Stage**				
I	1		1	
II	3.15 (1.12–8.88)	0.030	1.56 (0.47–5.19)	0.470
III	8.74 (3.21–23.79)	< 0.001	2.97 (0.96–9.12)	0.040
**Tumour site**				
Colon	1			
Rectum	0.97 (0.68–1.39)	0.881		
**Mor**–**index**				
Low	1		1	
High	0.49 (0.27–0.77)	0.003	0.21 (0.10–0.46)	< 0.001

*Abbreviation: OS* overall survival, *HR* hazard ratio, *CI* confidence interval, *Stage* tumor-node-metastasis, *Mor-index* Morisita-Horn ecological index

### Mor-index as an independent prognostic factor

**Fig. 6:**
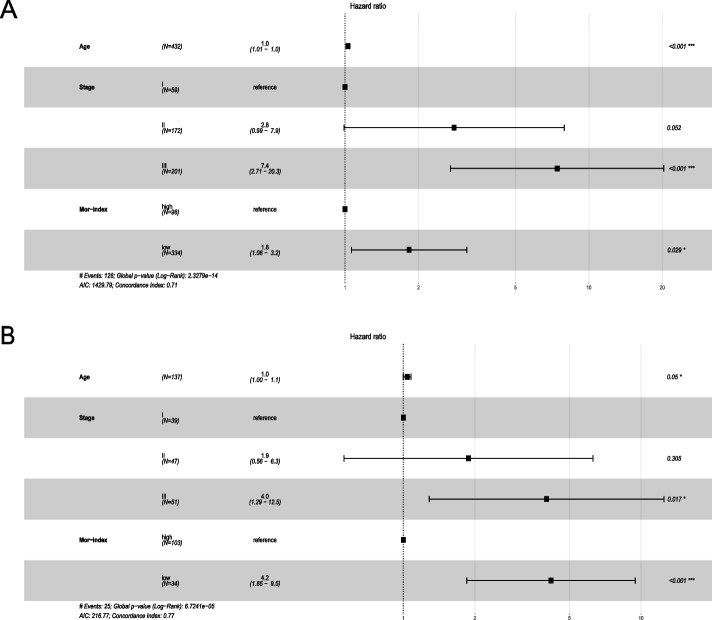
Forest plot represents multivariate Cox regression in the discovery cohorts and validation cohorts for overall survival

In Table 2, the univariate association between clinicopathological characteristics and OS is presented. We identified age, stage, and Mor-index as prognostic predictors for OS (*P* < 0.05). In multivariate analysis (Fig. 6), the Mor-index was still associated with OS, independent of age and stage. There was an association between a higher Mor-index and a better OS in the discovery cohort (discovery cohort: adjusted HR for low vs. high 1.80, 95% CI 1.06–3.20; *P* = 0.029; Fig. 6A) and validation cohort (4.20, 1.85–9.50; < 0.001; Fig. 6B).

## Discussion

As reported, a large number of immune cells interact closely with tumor cells, actively or negatively control the proliferation and death of tumor cells, and play a significant role in the invasion of tumor cells [22]. It is known that immune cells are associated with cancer treatment response and outcome, indicating the complexity of immune system involvement in cancer. However, the number of immune cells does not always predict the response to treatment, suggesting that other relevant factors may play a role [23]. Cancer cells can evade immune cells by evolving complex adaptive abilities, in the same way, that prey can evolve complex adaptations to evade predators [24]. Altogether, at the cellular level, the tumor environment of CRC is highly complex. The information proposed is insufficient if only tumor cells and immune cells are simply considered. Therefore, an indicator is needed to measure the interaction of these two types of cells, and the higher the value of the indicator, the better their degree of interaction.

The Morisita-Horn index exhibits the flexibility to adjust its exponents, allowing for the weighting of different factors [25]. This index is particularly suitable for analyzing populations with rare or abundant species, making it highly relevant for assessing the diverse data of immune and tumor cell densities. The Mor-index quantifies the association between tumor cells and immune cells in pathological sections, thus contributing to the advancement of research on the immune microenvironment in colorectal cancer (CRC). The colocalization of immune and tumor cells has been strongly associated with favorable prognosis, indicating the vital role of the immune system in recognizing, limiting, and impeding the survival, proliferation, and invasion of targeted cancer cells. Notably, previous discussions have demonstrated that higher Mor-index scores, indicating increased co-localization between tumor cells and immune cells, are predictive of a positive prognosis in breast cancer [10]. Subsequent studies consistently support the notion that a higher Mor-index is consistently linked to a more favorable prognosis in colorectal cancer (CRC). As depicted in Fig. 5, patients with a high Mor-index exhibited a good prognosis, whereas those with a low Mor-index had a poorer prognosis. Furthermore, the Mor-index emerged as an independent predictor, strengthening its significance as a prognostic indicator. These findings imply that the Mor-index holds potential as a reliable tool for predicting diverse survival outcomes in CRC patients.

This study obtained highly accurate nuclei segmentation results in WSIs of CRC. Nuclei image segmentation plays an essential role in medical diagnosis. The difficulties of nuclei segmentation in pathological images are blurred staining, uneven staining, adhesions between nuclei, differences in nuclei morphology, and high cost for the annotation. The Watershed method is a classical image segmentation method, which can obtain fast and accurate segmentation results through region growth segmentation [26, 27]. Based on the watershed segmentation algorithm, this study innovatively divided the image into tumor region, stroma region, and immune cell aggregation region, and then carries out nuclei segmentation and nuclei counting within the region, and eventually achieved an accuracy of 0.85. The results show that the algorithm speed and segmentation performance achieve the requirements. Meanwhile, quantifying the immune, tumor, and stroma nuclei in the immune microenvironment based on IHC staining of WSI, has been relatively little studied at this stage.

Malignant tumors are complex and represent physiological characteristics such as numerous spatial variations in gene expression and histopathology [28]. The mining of histopathological information by artificial intelligence technology is beneficial to the research of CRC. Overall, most studies generally use artificial intelligence technology to perform tissue segmentation and nuclear segmentation on HE-stained images to obtain valuable clinicopathologic indexes. There are few studies on tissue segmentation, nuclear segmentation, and classification for IHC-stained images. This study provided a novel approach to quantifying cells in tumor areas in IHC-stained images using artificial intelligence technology, which was helpful for clinical diagnosis. This study provided a novel approach to quantifying cells in tumor areas in IHC-stained images using artificial intelligence technology, which was helpful for clinical diagnosis.

However, there are certain limitations of this study. First, we used only one ecological score to evaluate the spatial association role of immune cells and tumor cells and their effects on the immune microenvironment. There are still plenty of other scoring options to explore. Another limitation is the number of validation centers, which are only validated in two centers and can be extended to more centers to validate our results. Furthermore, regarding the universality of the Mor-index, we believe that the Mor-index can have similar prognostic value in other cancers as well. However, more data will be needed in the future to validate it.

## Conclusion

This study introduced a novel AI-based method for accurately segmenting different types of nuclei within the tumor microenvironment (TME). The Mor-index, which reflects the immune status of colorectal cancer (CRC) patients, was found to be positively associated with favorable survival outcomes. These findings suggest that the Mor-index holds promise as a valuable tool for assisting in clinical prognosis and decision-making.

## Data Availability

The datasets used and/or analyzed during the current study are not publicly available due to personal information involved but are available from the corresponding author on reasonable request.
